# Inferring Incidence of Unreported SARS-CoV-2 Infections Using Seroprevalence of Open Reading Frame 8 Antigen, Hong Kong

**DOI:** 10.3201/eid3002.231332

**Published:** 2024-02

**Authors:** Shi Zhao, Chris Ka Pun Mok, Yun Sang Tang, Chunke Chen, Yuanxin Sun, Ka Chun Chong, David S.C. Hui

**Affiliations:** Author affiliations: School of Public Health, Tianjin Medical University, Tianjin, China (S. Zhao); JC School of Public Health and Primary Care, Chinese University of Hong Kong, Hong Kong, China (S. Zhao, C.K.P. Mok, Y.S. Tang, C. Chen, Y. Sun, K.C. Chong); Centre for Health Systems and Policy Research, Chinese University of Hong Kong, Hong Kong, China (S. Zhao, K.C. Chong); CUHK Shenzhen Research Institute, Shenzhen, China (S. Zhao, K.C. Chong); Li Ka Shing Institute of Health Sciences, Chinese University of Hong Kong, Hong Kong, China (C.K.P. Mok, Y.S. Tang, C. Chen, Y. Sun); S.H. Ho Research Centre for Infectious Diseases, Chinese University of Hong Kong, Hong Kong, China (C.K.P. Mok, D.S.C. Hui)

**Keywords:** COVID-19, viruses, SARS-CoV-2 Omicron variant, ORF8 antigen, infection attack rate, respiratory infections, zoonoses, Hong Kong, China

## Abstract

We tested seroprevalence of open reading frame 8 antigens to infer the number of unrecognized SARS-CoV-2 Omicron infections in Hong Kong during 2022. We estimate 33.6% of the population was infected, 72.1% asymptomatically. Surveillance and control activities during large-scale outbreaks should account for potentially substantial undercounts.

Hong Kong controlled the spread of COVID-19 caused by the SARS-CoV-2 Delta variant with stringent border control, effective contact tracing, and social distancing measures; only a small number of local SARS-CoV-2 infections had been reported in the 4 months before the Omicron variant appeared in late December 2021 (*1*). During the almost 2 years of pandemic before the Omicron outbreak, only ≈0.16% of the 7.5 million persons in Hong Kong were confirmed to be infected with SARS-CoV-2, among whom 200 persons died. An earlier investigation estimated that >99.5% of the population (>7 million persons) were naive to SARS-CoV-2 after the first 3 waves of community transmissions arising from imported cases (*2*). 

However, after the advent of the Omicron outbreak in Hong Kong, COVID-19 became uncontrolled in early 2022. The huge upsurge in cases, including daily COVID-19 death rates among the highest recorded globally, overwhelmed hospitals and led to an extreme shortage in critical care facilities (*3*). To maintain comprehensive disease surveillance, the government launched an online system for persons to self-report positive cases identified by self-administrated rapid antigen tests (RAT); RAT results were included in official surveillance reports beginning February 26, 2022 (*4*). Although reporting positive self-test results was compulsory in accordance with a local disease prevention and control ordinance, a large number of infections likely went untested and unreported because of a high proportion of asymptomatic or mild cases. 

Few empirical investigations have assessed the actual number of unrecognized infections during the Omicron epidemic, and estimates were mainly generated by modeling studies based on limited data. In previous studies, presence of open reading frame (ORF) 8 protein antibodies in blood samples was reported as a reliable serologic marker of natural SARS-CoV-2 infection (*5*,*6*). Given that ORF8 proteins are expressed only during the SARS-CoV-2 replication cycle, serologic testing for antibodies is able to determine whether a patient had been previously infected. 

In this study, we used the seroprevalence of ORF8 antigen antibodies to infer the actual number of unrecognized infections in an infection-naive population during the Omicron outbreak in Hong Kong. Our study was approved by the Joint Chinese University of Hong Kong/New Territories East Cluster Clinical Research Ethics Committee (ref no.: 2020.229). All participants who completed interview questionnaires or provided blood samples for this study signed informed consent forms. 

## The Study 

We obtained plasma samples from 1,028 volunteers >18 years of age during March 1–June 29, 2022, in the course of the Omicron BA.2 epidemic wave. All participants reported that, before the sampling date, they had never tested positive for COVID-19 by reverse transcription PCR test or RAT. We tested plasma samples using ELISA with ORF8 protein as an antigen for detection (Appendix). We obtained daily numbers of reported cases confirmed by PCR or RAT from the Hong Kong Department of Health. On the basis of the rates of ORF8 ELISA–positive test results relative to the number of reported cases at different time points, we estimated the true daily numbers of SARS-CoV-2 infections and infection attack rates by fitting a multinomial-distribution model, accounting for sensitivity and specificity of RAT and PCR tests. We assumed an initial infection attack rate of 0.2% before 2022, given Hong Kong’s stringent infection control measures before the Omicron outbreak (*2*,*7*). We also reconstructed the time-varying reproduction number by renewal equation (*8*). We used Markov chain Monte Carlo method to estimate the posterior distributions of model parameters, summarized by medians with 95% credible intervals (CrIs) (Appendix). 

Of the 1,028 self-reported uninfected persons in our study, 371 (36.1%) were male and 657 (63.9%) female, and median age was 50 (range 18–88) years; 1,027 reported having received >2 doses of COVID-19 vaccines. Overall positivity rate of ORF8 ELISA testing among our cohort was 2.5% (26 positive/1,028 tested). We found positivity rates were unlikely to vary on the basis of sex, age, or calendar date among self-reported uninfected persons in our cohort (Table). 

**Table:** Summary of testing status of SARS-CoV-2 ORF8 ELISA among 1,028 self-claimed uninfected persons, China, 2022*

Stratification	ORF8 test, no. ( %)		Positivity rate, %	p value†
	Positive	Negative		
Overall	26 (100)	1,002 (100)	2.5	NA
Sex				
F	12 (46.2)	645 (64.4)	1.8	0.062
M	14 (53.8)	357 (35.6)	3.8	
Age, y				
<40	6 (23.1)	305 (30.4)	1.9	0.715
40–65	16 (61.5)	566 (56.5)	2.7	
>65	4 (15.4)	131 (13.1)	3.0	
Test month				
March	2 (7.7)	97 (9.7)	2.0	0.608
April	2 (7.7)	137 (13.7)	1.4	
May	4 (15.4)	92 (9.2)	4.2	
June	18 (69.2)	676 (67.5)	2.6	

*NA, not applicable; ORF, open reading frame. †By 2-sided Fisher exact test.

Among the total population in Hong Kong, 16.2% were reported to have tested positive by RAT (6.1%) or reverse transcription PCR (10.1%). On the basis of estimates from our statistical model (Appendix), we inferred that 33.6% (95% CrI 32.1%–34.8%) of the 7.5 million persons in Hong Kong were infected during January 1–June 20, 2022 (Figure, panels A, B), corresponding to ≈2.5 million persons. We calculated percentages of asymptomatic cases of 41.8% among reported SARS-CoV-2 infections and 72.1% (95% CrI 70.8%–73.0%) among total (reported and unreported) infections. Reproduction numbers obviously dropped after positive RAT result reporting was implemented in Hong Kong on February 26, 2022 (Figure, panel C), consistent with findings about changes in transmission dynamics reported elsewhere (*9*). 

**Figure:**
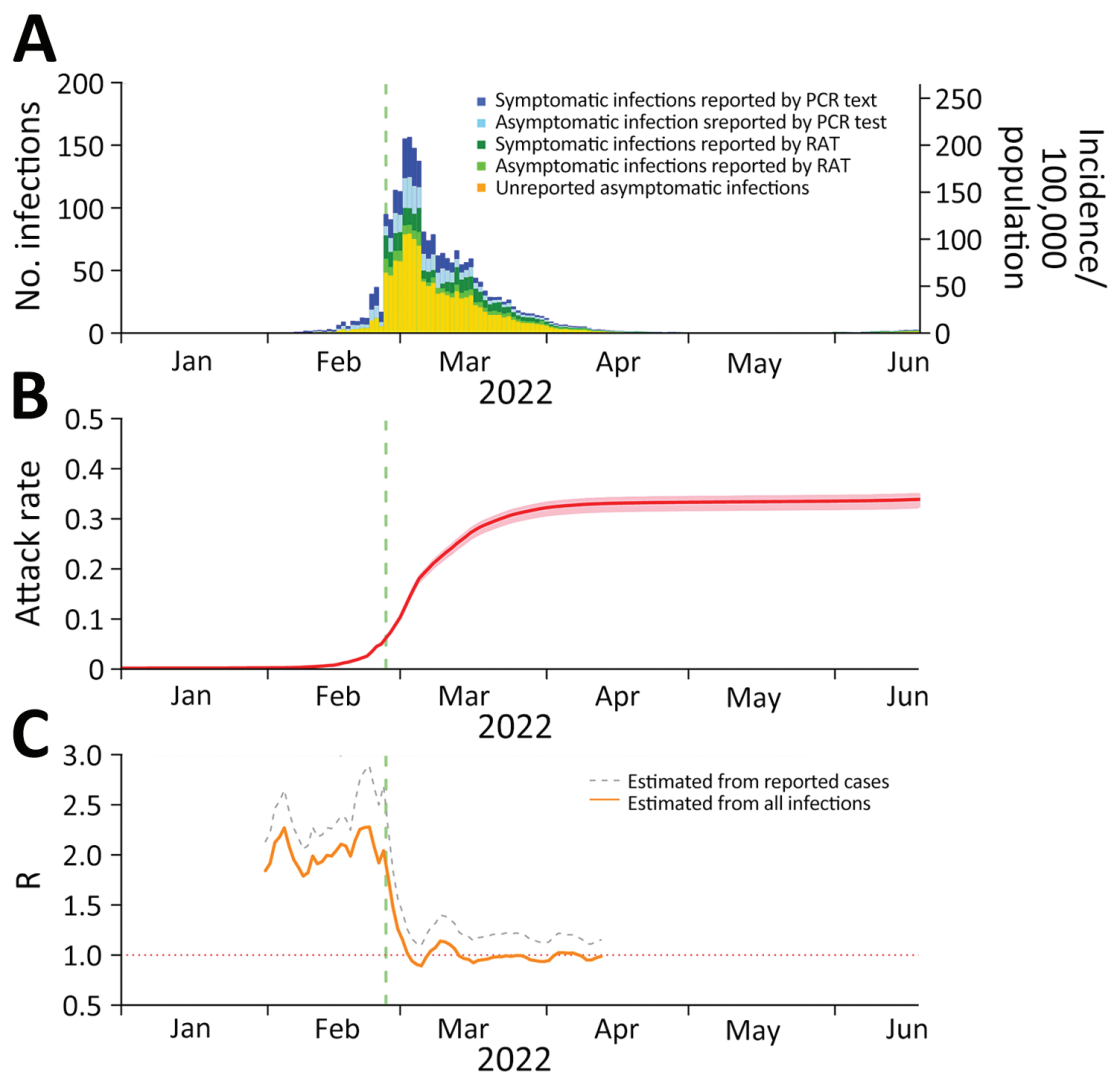
Reported SARS-COV-2 incidence versus estimates based on open reading frame 8 antigen testing, Hong Kong, China, January 1–June 20, 2022. A) Daily numbers and incidence of all reported infections and estimated asymptomatic infections by test type and presence or absence of symptoms. B) Estimated infection attack rate; shading indicates 95% credible intervals (CrIs). C) Estimated time-varying R for reported cases compared with estimated cases. Green vertical dashed lines indicate date (February 26, 2022) when compulsory reporting of positive RAT results was implemented in Hong Kong. Because of the large number of cases, 95% CrIs for R were extremely narrow, and thus we omitted CrIs in panel C. R, reproduction number; RAT, rapid antigen test.

## Conclusions 

Using the seroprevalence of ORF8 antigens, we inferred that 16.2% of 33.6% (≈1/2) SARS-CoV-2 infections during the Omicron epidemic in Hong Kong were unrecognized, despite RATs being widely disseminated and reporting of positive results made locally compulsory. Our estimates of asymptomatic proportions were generally higher than estimates previously reported for earlier variants (*10*). With such a large number of unrecognized cases circulating the virus in the community, it was not surprising that the Omicron outbreak was uncontrollable, even though stringent measures, such as contact tracing and quarantine for close contacts, continued to be in effect. Our study findings highlight the usefulness of testing for ORF8 seroprevalence among efforts to monitor COVID-19 outbreaks, especially for emerging new variants of concern. Public health agencies need to take into account the potential for substantial undercount of actual numbers of infections when considering the commitment of resources to prevent and control outbreaks. 

Appendix Additional information about a study of estimating actual SARS-COV-2 incidence in Hong Kong, 2022.
