# Supplementary material for: Inferring Incidence of Unreported SARS-CoV-2 Infections Using Seroprevalence of Open Reading Frame 8 Antigen, Hong Kong
Source: Emerg Infect Dis. 2024 Feb;30(2):325–8. doi: 10.3201/eid3002.231332 (PMC10826773; doi:10.3201/eid3002.231332)
Supplement: Appendix — Additional information about a study of estimating actual SARS-COV-2 incidence in Hong Kong, 2022. [file 23-1332-Techapp-s1.pdf]

Article DOI: https://doi.org/10.3201/eid3002.231332

EID cannot ensure accessibility for Appendix materials supplied by authors. Readers who have difficulty accessing Appendix content should contact the authors for assistance.

Inferring Incidence of Unreported SARS-CoV-2 Infections Using Seroprevalence of Open Reading Frame 8 Antigen, Hong Kong

Appendix

Table of Contents

1 Laboratory methods **2**

1.1 Specimen collection 2

1.2 Protein expression and purification 2

1.3 ELISA binding assay 2

2 Statistical modelling **3**

2.1 Model specification 3

2.2 Likelihood framework 3

2.3 Parameter estimation 5

3 Extended discussion on limitations **5**

Supplementary references **6**

1 Laboratory methods

1.1 Specimen collection

Specimens of clotted blood were collected from the patients. The serum was separated from the blood by centrifugation and stored at –80°C until use. Approval for the study was obtained from the Joint Chinese University of Hong Kong-New Territories East Cluster Clinical Research Ethics Committee (ref no.: 2020.229).

1.2 Protein expression and purification

The full length ORF8 protein of SARS-CoV-2 was fused with a C-terminal His6 tag and was cloned into a customized pCAGEN vector. The ORF8 protein was expressed by transfecting purified plasmid into Expi293 cells using Gibco Expi293 Fectamine Transfection kit. The cells were incubated in a shaker at the condition of 8% CO_2, and 37ºC for 6 days. The ORF8 protein from the supernatant was then purified by Ni-NTA and concentrated using a 3K Protein concentrator.

It has been shown that SARS-CoV-2 contains a unique ORF8 accessory gene that is absent from other known human pathogenic coronaviruses (*1*). In our previous study (*2*), we found that ORF8, as a non-structural protein, was only expressed in COVID-19 patients, and it was highly immunogenic when compared to other ORF and structural proteins. Thus, seropositive to ORF8 antigen provides evidence of being infected by SARS-CoV-2, and this approach has been used by us and others (*3*,*4*).

1.3 ELISA binding assay

The 96-well enzyme-linked immunosorbent assay (ELISA) plates (Nunc MaxiSorp, Thermo Fisher Scientific) were first coated overnight with 100 ng per well of purified recombinant protein in PBS buffer. The plates coated with the purified recombinant protein were then blocked with 100ul of Chonblock Blocking/Sample Dilution ELISA Buffer (Chondrex, Inc, USA) at room temperature for 2 hours. Each serum or plasma sample was tested at a dilution of 1:100 in Chonblock Blocking/Sample Dilution ELISA Buffer and 100ul of diluted sample was added to the ELISA wells of each plate for 2-hour incubation at 37°C. After extensive washing with PBS containing 0.1% Tween 20, HRP-conjugated goat anti-human IgG (1:5000, GE Healthcare) was added for 1 hour at 37°C. The ELISA plates were then washed five times with PBS containing 0.1% Tween 20. Subsequently, 100 μL of HRP substrate (Ncm TMB One) (New Cell & Molecular Biotech Co. Ltd, China) was added into each well. After 15 minutes incubation, the reaction was stopped by adding 50 μL of 2 M H_2SO_4 solution and analyzed on an absorbance microplate reader at 450 nm wavelength.

The assay was initially validated using 100 negative controls and 100 convalescent sera from adults in Hong Kong who had recovered from Omicron infection one month prior. We defined a serum to be positive for ORF8 antigen if the OD value was 3 standard deviations (SD) above the mean of the negative controls, which in our assay was 0.28.

2 Statistical modelling

2.1 Model specification

We consider an individual who was infected by SARS-CoV-2 in period from January 1 to June 20, 2022 can be classified into six mutually exclusive and exhaustive types according to the detection and reporting status. These included the follows

Detected and reported

- Type (#1): correctly detected by PCR test with test sensitivity s_{PCR} and 100% reported,
- Type (#2): correctly detected by RAT with test sensitivity s_{RAT} and reported with self-reporting ratio r_{RAT},

Detected and unreported

- Type (#3): correctly detected by RAT with test sensitivity s_{RAT} and unreported with ratio $1 - r_{\mathrm{RAT}}$,

Undetected and unreported

- Type (#4): undetected by PCR test with false negative ratio $1 - s_{\mathrm{PCR}}$ and 100% unreported,
- Type (#5): undetected by RAT with false negative ratio $1 - s_{\mathrm{RAT}}$ and 100% unreported,
- Type (#6): never receive any test and thus 100% unreported.

Here, according to the type of test received for each SARS-CoV-2 infection, the six types above can be re-arranged into the following three classes including

- Class (#1): infection who received PCR test, i.e., types (#1) and (#4),
- Class (#2): infection who received RAT, i.e., types (#2), (#3) and (#5),
- Class (#3): infection who never received any test, i.e., type (#6).

In each class above, the distribution of composed types can be determined given the information of s_{PCR}, s_{RAT}, and r_{RAT}. From existing literature, we set s_{PCR} at 99.0%, and s_{RAT} at 81.0%. Fort the specificity, considering that both RT-PCR test and RAT have high level of specificity (> 99.9%) for detecting SARS-CoV-2 infection, we set specificity to be 100%, which

would unlikely to change the modelling results but saved a large level of model complexity. Different values of r_{RAT} could be considered in further analysis.

We assume the probability for each infection falls in any of the three classes as a categorical (i.e., multinoulli) distribution with three parameters $p_{\mathrm{PCR}} = p_1$ for class (#1), $p_{\mathrm{RAT}} = p_2$ for class (#2), and $p_{\mathrm{no}} = p_3 = 1 - (p_1 + p_2)$ for class (#3), where all parameters range from 0 to 1 with sum of 1. Specially, from January 1 to February 25 (i.e., week 1 to week 8), 2022, before the implementation of mass RAT testing in Hong Kong, we consider the categorical distribution as $p_{\mathrm{PCR}} = p_1$ for class (#1), $p_{\mathrm{RAT}} = 0$ for class (#2), and $p_{\mathrm{no}} = p_2 + p_3 = 1 - p_1$ for class (#3).

2.2 Likelihood framework

To estimate the daily number of SARS-CoV-2 infections, $a_{\mathrm{all}}(t)$, and parameters p_1, p_2 and r_{RAT}, we construct the following two likelihood functions. The likelihood functions were defined and thus can be updated on a daily basis, so that the depletion of susceptible population was accounted.

Likelihood for observing $c_{\mathrm{PCR}}(t)$ cases reported by PCR test and $c_{\mathrm{RAT}}(t)$ cases reported by RAT was a multinomial distribution as $\mathrm{Multinomial}\big(\boldsymbol{x} = \big[c_{\mathrm{PCR}}(t), c_{\mathrm{RAT}}(t), \big(a_{\mathrm{all}}(t) - c_{\mathrm{PCR}}(t) - c_{\mathrm{RAT}}(t)\big)\big], \mathrm{size} = a_{\mathrm{all}}(t), \mathrm{probabilities} = [p_{\mathrm{PCR}} s_{\mathrm{PCR}}, p_{\mathrm{RAT}} s_{\mathrm{RAT}} r_{\mathrm{RAT}}, (1 - p_{\mathrm{PCR}} s_{\mathrm{PCR}} - p_{\mathrm{RAT}} s_{\mathrm{RAT}} r_{\mathrm{RAT}})]\big)$.

Likelihood for observing $x(t)$ test-positive individuals among $m(t)$ random-selected individuals who claim never being test positive on or before the *t*-th day was a hypergeometric distribution. The distribution describes the probability of $x(t)$ test-positive individuals in $m(t)$ random-selected individuals, without replacement, from a finite population (who claim never being test positive) of size $N \cdot [1 - \mathrm{IAR}(0) - (\mathrm{IAR}(t) - \mathrm{IAR}(0)) \cdot (p_{\mathrm{PCR}} s_{\mathrm{PCR}} + p_{\mathrm{RAT}} s_{\mathrm{RAT}})]$ that contains exactly $N \cdot (\mathrm{IAR}(t) - \mathrm{IAR}(0)) \cdot [1 - (p_{\mathrm{PCR}} s_{\mathrm{PCR}} + p_{\mathrm{RAT}} s_{\mathrm{RAT}})] \cdot s_{\mathrm{TEST}}$ individuals that were supposed to have a test-positive status but unaware by themselves (if a test were arranged). By contrast, there were $N \cdot \big(1 - \mathrm{IAR}(t)\big) + N \cdot (\mathrm{IAR}(t) - \mathrm{IAR}(0)) \cdot [1 - (p_{\mathrm{PCR}} s_{\mathrm{PCR}} + p_{\mathrm{RAT}} s_{\mathrm{RAT}})] \cdot (1 - s_{\mathrm{TEST}})$ were supposed to have a test-negative status if a test were arranged. The hypergeometric distribution can be expressed as $\mathrm{Hypergeometric}\big(x = x(t), \text{sampling size} = m(t), \text{total positive} = N \cdot \big(\mathrm{IAR}(t) - \mathrm{IAR}(0)\big) \cdot [1 - (p_{\mathrm{PCR}} s_{\mathrm{PCR}} + p_{\mathrm{RAT}} s_{\mathrm{RAT}})] \cdot s_{\mathrm{TEST}}, \text{total population} = N \cdot [1 - \mathrm{IAR}(0) - (\mathrm{IAR}(t) - \mathrm{IAR}(0)) \cdot (p_{\mathrm{PCR}} s_{\mathrm{PCR}} + p_{\mathrm{RAT}} s_{\mathrm{RAT}})]\big)$.

Here, N denoted the fixed population size, which was 7.5 million in Hong Kong,

$\mathrm{IAR}(t)$ denoted the infection attack rate with range from 0 to 1, s_{TEST} denoted the test sensitivity of the laboratory test (i.e., ORF8 test) used for samples collected in this study, and

s_{TEST} was fixed at 75%. Among 100 negative controls (from collected external source, data not shown) who were free of SARS-CoV-2 infection and were used to check the specificity of ORF8 test, we detected 0 false-positive subject, and thus we set the specificity of ORF8 test in this model to be 100%.

The initial value of attack rate $\mathrm{IAR}(0)$ was fixed at 0.2% accounting the attack rate during COVID-19 outbreaks before 2022. As such, we have $\mathrm{IAR}(t) = \mathrm{IAR}(0) + \sum_{\tau \le t} a_{\mathrm{all}}(\tau) / N$.

2.3 Parameter estimation

The parameters to be estimated were daily number of SARS-CoV-2 infections, $a_{\mathrm{all}}(t)$, and p_1, p_2 and r_{RAT}. We combined the two likelihood functions, i.e., multinomial distribution for cases reported by PCT test or RAT and hypergeometric distribution for the samples among those who were unaware of their testing status, so that all parameters can be estimated simultaneously. We adopted a Bayesian fitting procedure with Metropolis-Hastings algorithm, which was a Markov chain Monte Carlo (MCMC) method, with non-informative prior distributions for parameter estimation. The MCMC method was practiced with 10 chains and 100,000 iterations for each chain, including 40,000 iterations for the burn-in period, to obtain the posterior estimates. The convergence of each MCMC chain was visually checked using trace plots and the Gelman–Rubin–Brooks diagnostic quantitatively (*5*). The median and 95% credible intervals (95% CrI) of the posterior distributions of model parameters were calculated for summary.

3 Extended discussion on limitations

This study has limitations.

First, among the samples of 1028 self-claimed uninfected individuals recruited in this study, female ratio (63.9%) was higher than the situation of Hong Kong population (54.3% in 2022 from the Census and Statistics Department). This disproportion of females is likely due to female individuals are generally more willing to participate in survey activities related to health, which leads to a higher proportion of females in our samples. However, according to the literature about the COVID-19 epidemic situation in Hong Kong (*6*,*7*), the association between sex and risk of SARS-CoV-2 infection is unlikely. Therefore, the disproportional females in our 1028 samples are unlikely to bias our estimation of overall infection attack rate of Hong Kong population, because both sexes are likely to have the same infection attack rate (as well as risk of infection).

Second, initial infection attack rate $\mathrm{IAR}(0)$ at 0.2% was based on real-world situation of COVID-19 epidemic in Hong Kong from January 2020 to December 2021, under the

background of the previous "zero-COVID" policy. We noted that the cases in Hong Kong were reported with high infection-detecting efforts, contact tracing and disease control intensity before 2022, and large-size of cases number was unlikely to occur. Although it is difficult to have information on the exact value of initial infection attack rate, and slight changes in this setting is unlikely to affect our main findings. In addition, we neglected the re-infection scenario for this 0.2% of the population, which simplified the analysis and had minor impact to the IAR estimates.

References

1. Chan JF, Kok KH, Zhu Z, Chu H, To KK, Yuan S, et al. Genomic characterization of the 2019 novel human-pathogenic coronavirus isolated from a patient with atypical pneumonia after visiting Wuhan. Emerg Microbes Infect. 2020;9:221–36. PubMed https://doi.org/10.1080/22221751.2020.1719902

2. Hachim A, Kavian N, Cohen CA, Chin AWH, Chu DKW, Mok CKP, et al. ORF8 and ORF3b antibodies are accurate serological markers of early and late SARS-CoV-2 infection. Nat Immunol. 2020;21:1293–301. PubMed https://doi.org/10.1038/s41590-020-0773-7

3. Wang X, Lam JY, Wong WM, Yuen CK, Cai JP, Au SW, et al. Accurate diagnosis of COVID-19 by a novel immunogenic secreted SARS-CoV-2 orf8 protein. mBio. 2020;11:e02431-20. PubMed https://doi.org/10.1128/mBio.02431-20

4. Huang J, Zhao S, Chong KC, Zhou Y, Lu W, Fang F, et al. Infection rate in Guangzhou after easing the zero-COVID policy: seroprevalence results to ORF8 antigen. Lancet Infect Dis. 2023;23:403–4. PubMed https://doi.org/10.1016/S1473-3099(23)00112-3

5. Gelman A, Carlin JB, Stern HS, Dunson DB, Vehtari A, Rubin DB. Bayesian data analysis. Boca Raton, Florida: CRC Press; 2013.

6. Tsang NNY, So HC, Cowling BJ, Leung GM, Ip DKM. Effectiveness of BNT162b2 and CoronaVac COVID-19 vaccination against asymptomatic and symptomatic infection of SARS-CoV-2 omicron BA.2 in Hong Kong: a prospective cohort study. Lancet Infect Dis. 2023;23:421–34. PubMed https://doi.org/10.1016/S1473-3099(22)00732-0

7. Yang B, Tsang TK, Gao H, Lau EHY, Lin Y, Ho F, et al. Universal community nucleic acid testing for coronavirus disease 2019 (COVID-19) in Hong Kong reveals insights into transmission dynamics: a cross-sectional and modeling study. Clin Infect Dis. 2022;75:e216–23. PubMed https://doi.org/10.1093/cid/ciab925
